# Complex soft tissue and facial bone reconstruction after a bear attack in Indonesia: a case report of definitive management following an 8-hour interfacility transfer

**DOI:** 10.1093/jscr/rjag114

**Published:** 2026-03-07

**Authors:** Amira Azra Arisa Putri, Mufida Muzakkie

**Affiliations:** Plastic Reconstructive and Aesthetic Surgery Division, Mohammad Hoesin Central General Hospital - Faculty of Medicine, Sriwijaya University, Jl. Jenderal Sudirman Km. 3.5, Sekip Jaya, Kemuning District, Palembang 30126, Indonesia; Plastic Reconstructive and Aesthetic Surgery Division, Mohammad Hoesin Central General Hospital - Faculty of Medicine, Sriwijaya University, Jl. Jenderal Sudirman Km. 3.5, Sekip Jaya, Kemuning District, Palembang 30126, Indonesia

**Keywords:** bear attack, maxillofacial trauma, facial reconstruction, resource-limited setting

## Abstract

Bear attacks, although rare, may result in severe maxillofacial trauma, highlighting the devastating consequences of human-wildlife conflict in rural areas. Bear maulings typically cause cutting, penetrating, and crushing injuries, with most victims sustaining contaminated maxillofacial trauma requiring comprehensive management to prevent complications. We report a case of a 68-year-old woman presenting with sustained severe left-sided facial trauma following a bear attack. The patient had a full-thickness hemifacial avulsion, fractures of the zygomaticum and maxilla, and a non-salvageable ocular injury after an 8-hour transfer. Management included debridement, wire fixation of fractures, ocular exenteration, and anatomically re-approximated soft-tissue closure. This case highlights the importance of early trauma principles, effective infection control, and tailored fixation strategies for fragile facial bones. It emphasizes the need for staged reconstruction planning in resource-limited and geographically distant settings. Integrated multidisciplinary management and meticulous surgical decision-making are essential for achieving functional and aesthetic recovery in such complex injuries.

## Introduction

In many developing countries, including in Asia, agriculture and livestock provide livelihoods for large populations, placing communities near forested areas at increased risk of human–wildlife conflict, including bear encounters [1]. Bear mauling cause injuries ranging from cutting and penetrating wounds to severe crushing injuries [2]. Nearly 90% of victims sustain maxillofacial fractures, heavy bleeding, and tissue avulsion, often complicated by contamination from the bear’s oral flora, claws, soil, and organic debris [2, 3]. Such wounds demand complex debridement and reconstruction to restore form and function, prevent complications, and optimize post-traumatic outcomes [4].

Pagar Alam in South Sumatra, Indonesia, a major coffee-producing region, faces significant risk of life-threatening head and facial injuries from wildlife encounters [1, 5]. Patients with severe maxillofacial injuries are often referred to Palembang, the sole provincial center with Plastic Surgery services; however, long transport times can delay care and heighten infection risk. Life-threatening hemorrhage from maxillofacial trauma can be fatal and necessitates urgent intervention [6, 7]. Data on bear attacks in Indonesia remain limited; this case report describes complex facial reconstruction following a bear mauling, highlighting challenges in initial care and delayed surgery in resource-limited settings.

## Case description

A 68-year-old woman was referred from Pagar Alam to Mohammad Hoesin Hospital in Palembang following a bear attack on a coffee plantation adjacent to a wilderness area. She sustained severe maxillofacial trauma after being clawed, dragged, and rolled ~10 m. The inter-facility transfer lasted 8 hours, during which she received ringer’s lactate, intravenous ceftriaxone, ketorolac, tranexamic acid, and a pressure bandage for hemostasis, with continuous monitoring for neurological deterioration. She remained conscious throughout, with no nausea, vomiting, or seizures.

**Figure 1:**
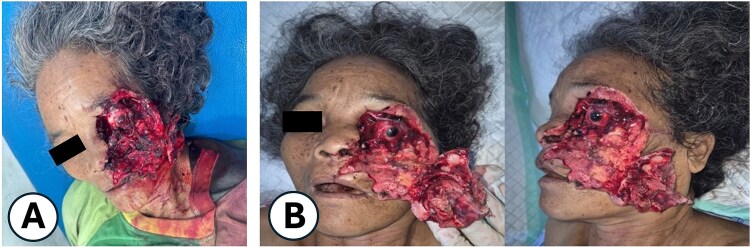
Clinical presentation of the patient after a bear mauling. (A) Initial condition upon arrival at Pagar Alam District Hospital. (B) Condition upon admission to Dr. Mohammad Hoesin Hospital as the referral center.

**Figure 2:**
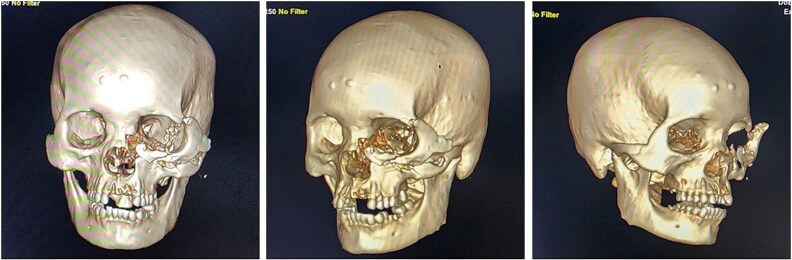
CT scan findings of the patient after a bear mauling.

Upon arrival, the patient was alert, haemodynamically stable, with primary survey within normal limits. Secondary survey revealed a full-thickness left hemifacial avulsion, comminuted fractures of the zygomatic and maxillary bones, complete avulsion of the upper central incisors, and severe left ocular destruction involving complete injury to the extraocular muscle and lacrimal system, resulting in loss of vision and motility (Figs 1 and 2). Early management included administration of rabies vaccination (Verorab), anti-tetanus serum, and tetanus toxoid.

**Figure 3:**
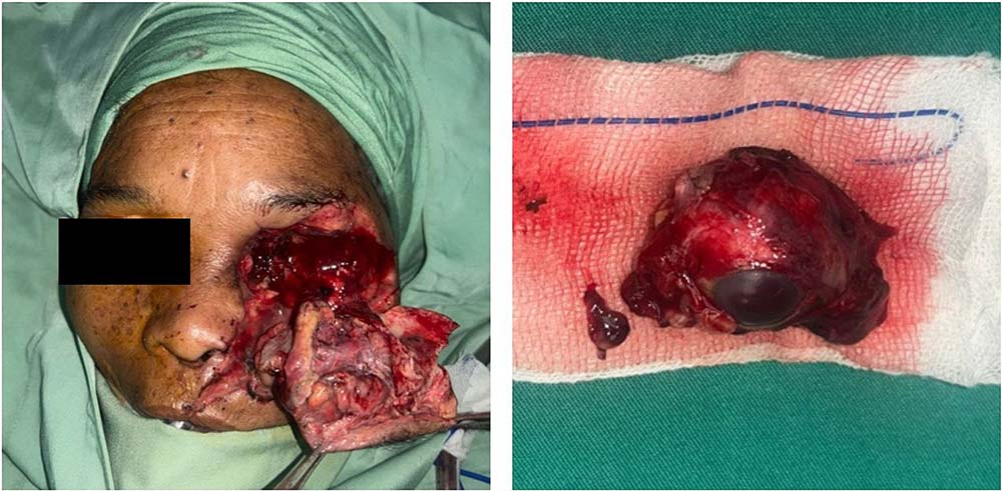
Intraoperative appearance of the patient following exenteration.

**Figure 4:**
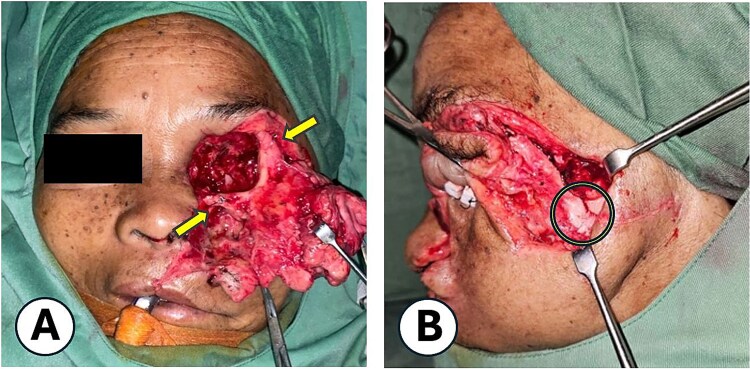
Internal fixation technique. (A) Application of wire for stabilization of the midfacial fracture, with fixation at the zygomaticofrontal bone and lower orbital rim (maxilla). (B) Anatomical realignment of the fracture segments. Following wire fixation, proper alignment of the zygomatic arch was achieved.

**Figure 5:**
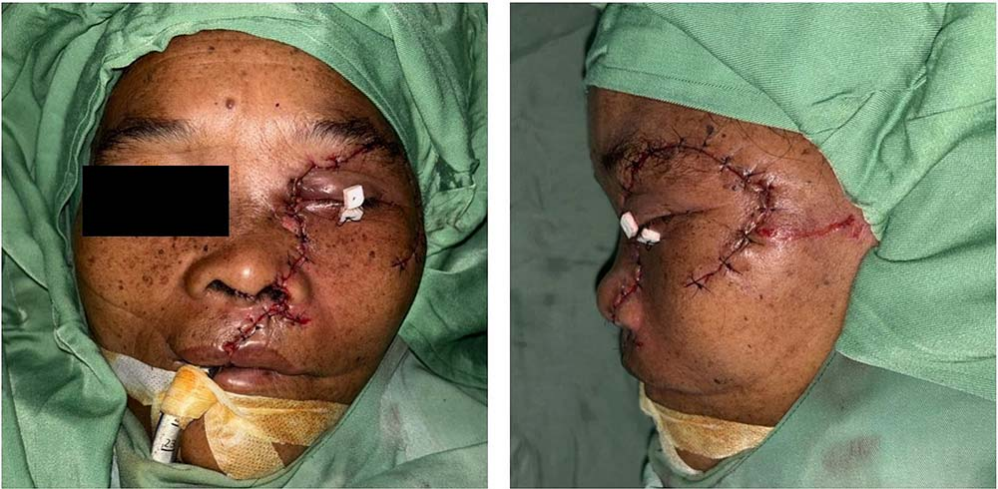
Immediate postoperative outcome. Anatomical repositioning and puzzle-like soft-tissue re-approximation were performed after debridement, exenteration, and fracture fixation.

Under general anesthesia, the highly contaminated wound was thoroughly debrided. The Ophthalmology team performed a left orbital exenteration (Fig. 3), followed by reconstructive surgery by the Plastic Surgery team. Internal wire fixation stabilized fractures of the frontal process of the zygomatic bone and lower infraorbital rim (maxilla), while the zygomatic arch was realigned without fixation due to fragility (Fig. 4). Avulsed soft tissues were repositioned and re-approximated. The subcutaneous layers were closed with 4–0 polydioxanone, and skin closure was achieved using 5–0 polypropylene (Fig. 5). On postoperative Day 2, purulent orbital discharge indicated infection; culture was performed, but allergies limited antibiotic options, so ceftriaxone was continued. The second and third doses of Verorab were administered on postoperative Days 3 and 7. The patient was discharged on Day 8 with a prescribed 7-day course of oral antibiotics.

**Figure 6:**
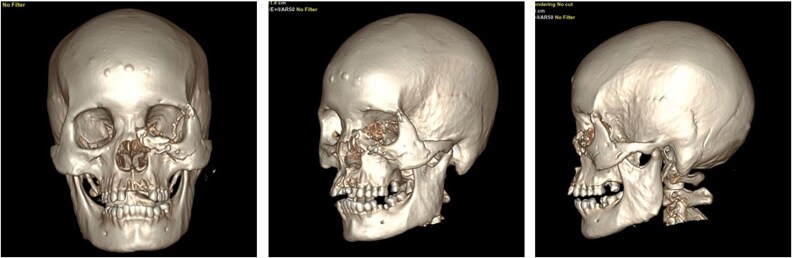
Postoperative CT evaluation demonstrating successful bony consolidation and stable wire fixation.

**Figure 7:**
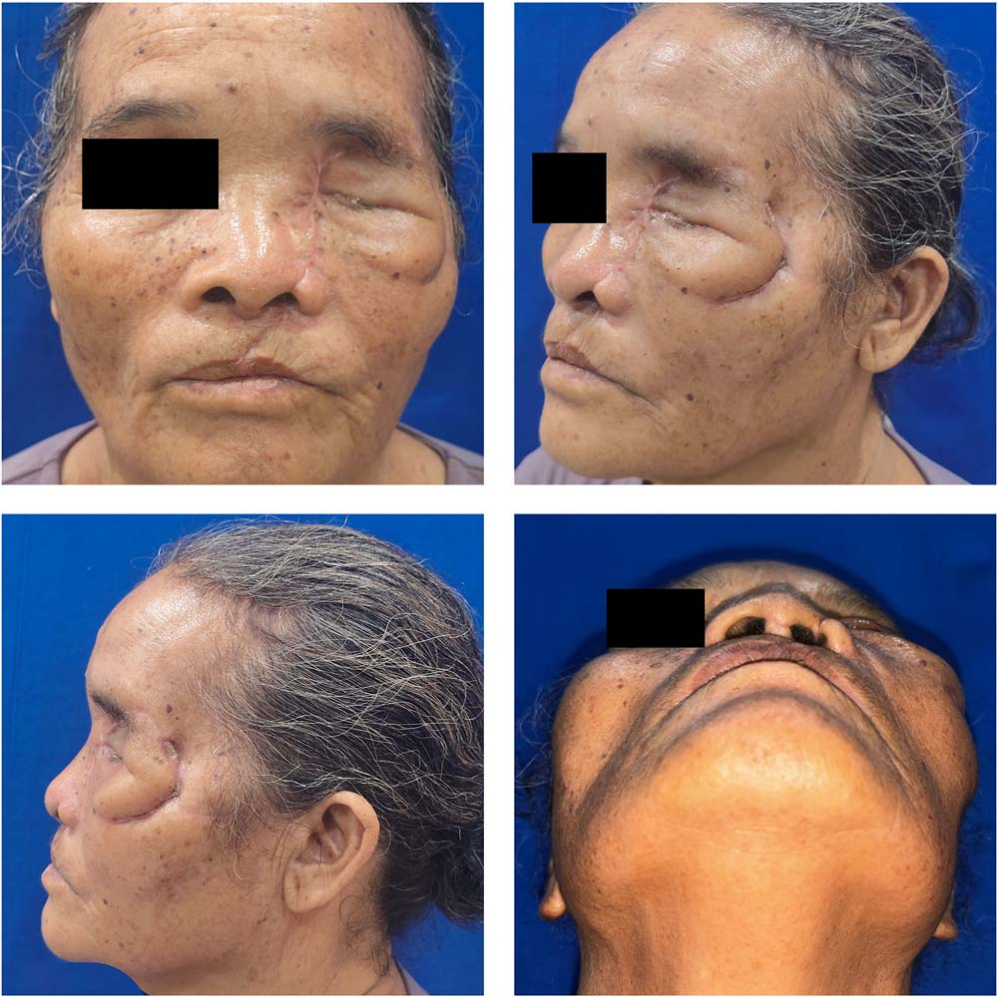
Clinical condition at the 4-month postoperative follow-up.

At 2-week follow up, the wound showed satisfactory healing with minimal edema, allowing suture removal, and the fourth Verorab dose was given. By 6 weeks, CT imaging confirmed stable fixation and fracture union (Fig. 6), and at the 4-month postoperative follow-up, the wound had fully healed, with restored normal mastication and speech function (Fig. 7).

## Discussion

Management of severe maxillofacial trauma from bear attacks requires strict adherence to trauma principles. Although the patient was initially stable, such injuries may rapidly deteriorate due to edema, hemorrhage, or airway compromise. The literature emphasizes prioritizing airway management, hemodynamic stabilization, and hemorrhage control before reconstruction [4, 6, 8].

Midfacial fractures carry high hemorrhage risk due to rich vascularity [8]. In this case, hemorrhage was controlled with direct pressure and bandaging, the most reliable non-invasive method during transfer [3]. Temporary suturing was avoided to prevent premature closure of contaminated wounds, which could exacerbate infection and compromise outcomes.

Definitive skeletal treatment must account for fragment fragility. Open reduction with plates and screws was avoided due to potential iatrogenic fragmentation and infection [2, 5]. Wire fixation was chosen as it offers adequate stability, minimizes tissue damage, usually does not require removal, and is suitable for contaminated wounds since smooth stainless-steel wires harbour fewer bacteria than plated implants. Figure-of-eight constructs provided semi-rigid support appropriate for fractures without major bone loss. Reports of bear-related facial fractures demonstrated satisfactory results with similar methods, supporting wire fixation as a safer and cost-efficient option in limited-resource settings [9, 10].

Following debridement and fracture stabilization, avulsed soft-tissue segments were anatomically re-approximated in a ‘puzzle-like’ configuration to restore facial contours. In severe avulsive injuries with viable tissues, anatomical repositioning is preferred over primary closure because it preserves native tissue, reduces distortion, and improves long-term outcomes [11]. Subcutaneous closure employed 4–0 polydioxanone (synthetic absorbable monofilament) sutures offering a lower infection risk and superior strength, while skin closure employed 5–0 polypropylene (synthetic non-absorbable monofilament) sutures due to their low tissue reactivity and favourable cosmetic results [7, 12].

Adjunctive pharmacologic management included analgesics, broad-spectrum antibiotics, and rabies and tetanus prophylaxis. Despite these measures, infection risk remained high due to contamination, limited vascularity, wire-associated biofilm potential, and poor wound hygiene in rural settings—consistent with other bear-related trauma cases [2, 5].

A 2-week course of prophylactic antibiotics was warranted due to high contamination risk [2]. In allergic patients, antibiotic selection depends on alternatives that provide anaerobic coverage. Tetanus prophylaxis was mandatory, as all mauling wounds are susceptible to tetanus infection. Administration of tetanus vaccine and human tetanus immunoglobulin is recommended for contaminated wounds with uncertain immunization status, offering immediate passive immunity [13]. Rabies prophylaxis was also essential; Verorab was administered in a four-dose regimen (Days 0, 3, 7, and 14) as post-exposure prophylaxis for unvaccinated patients [14].

Given the long referral distance, discharge education was emphasized, with clear instructions on wound care, infection signs, hardware exposure, and the importance of strict follow-up, as structured education has been shown to improve outcomes in remote trauma patients [4]. Long-term management focused on functional and aesthetic rehabilitation, including staged orbital socket reconstruction to enable prosthesis placement, restoring facial symmetry and psychological well-being. Scar optimization was planned post-healing, supported by evidence favouring silicone gels or sheets, corticosteroid injections, and laser therapy to improve scar colour, texture, and pliability [15].

## Data Availability

All data generated or analysed during this study are included in this article. Additional data are available upon reasonable request.
